# Immunity against reinfection in pigs following Taenia solium infection and a quantitative dose-response model

**DOI:** 10.21203/rs.3.rs-6496988/v1

**Published:** 2025-04-23

**Authors:** Eloy Gonzales-Gustavson, Francesco Pizzitutti, Gabrielle Bonnet, Claudio Muro, Ricardo Gamboa, Javier A. Bustos, Sarah Gabriël, William K. Pan, Héctor H. Garcia, Seth ÓNeal

**Affiliations:** Universidad Nacional Mayor de San Marcos; Universidad San Francisco de Quito; London School of Hygiene & Tropical Medicine; Universidad Peruana Cayetano Heredia; Universidad Peruana Cayetano Heredia; Universidad Peruana Cayetano Heredia; Ghent University; Duke University; Universidad Peruana Cayetano Heredia; Oregon Health & Science University and Portland State University

**Keywords:** Taenia solium, experimental infection, acquired immunity, reinfection, quantitative dose-response, live cysts prediction

## Abstract

**Background:**

*Taenia solium* is a zoonotic parasite causing significant health and economic burdens, with complex transmission dynamics requiring improved control strategies.

**Methods:**

This study investigates the effect of *T. solium*infection and reinfection on cyst development in pigs and evaluates how acquired immunity constrains parasite burden. A total of 116 pigs were purchased from commercial farms in northern Peru and housed in pathogen-free facilities under controlled conditions. Of these, 110 pigs were allocated to 18 experimental groups to (1) evaluate the impact of infection and reinfection with varying doses of *T. solium* eggs and (2) generate a model to predict the number of live cysts produced, given the dose and age at infection. Gravid proglottids collected from human cases of *T. solium* taeniasis were used to prepare egg pools, ensuring viability consistency. Infections were administered orally using gelatin capsules via esophageal catheterization, followed by necropsy 10 weeks after the final infection event to quantify cysts. A negative binomial regression model was used to analyze cyst burden dependence on infection dose, past infection, age, and other factors.

**Results:**

No statistically significant differences in cyst counts were observed between pigs infected once and those that were reinfected, regardless of the initial dose (as low as 100 eggs) or reinfection dose (up to 20,000 eggs). This finding highlights that infection results in strong acquired immunity, effectively blocking subsequent infections. A quantitative dose-response model suggests that the relationship between egg dose and the number of viable cysts is best described by a power relationship. Combining data from single-infection and reinfected pigs into a unified model improved prediction precision. Finally, incorporating age at infection results in a model of the number of viable cysts in pigs depending on dose and age that combines acquired and innate immunity effects, i.e. changes in susceptibility with age.

**Conclusions:**

Initial exposure to *T. solium*eggs induces strong acquired immunity in pigs, effectively preventing reinfection. Our quantitative dose-response model predicting live cyst counts based on egg dose and pig age offers valuable insights for integrating immunity processes into models of *T. solium* transmission.

## Background

*Taenia solium* is a zoonotic parasite causing an estimated 2.8 million disability-adjusted life years (DALYs) globally [1]. It also leads to significant economic losses, particularly in low- and middle-income countries [2]. The parasite is listed among the 20 diseases that disproportionately affect marginalized populations in tropical and subtropical regions and are targeted for control, elimination, or eradication by 2030 [3]. Humans serve as the definitive host, harboring the adult tapeworm, which sheds proglottids and eggs in feces. These eggs are ingested by the intermediate host, the pig, in which cysts develop in muscle and other tissues. Humans can also act as accidental intermediate hosts when they ingest eggs, sometimes leading to the development of neurocysticercosis, a severe condition characterized by cysts in the central nervous system [4].

Understanding the dynamics of transmission—how, when, where, and why pigs become infected with different numbers of cysts in rural areas—is critical for designing effective control strategies [5]. One of the challenges relates to understanding the high variability in the number of cysts in infected pigs, with most infections involving fewer than 10 cysts (making them often hard to identify through meat inspection) while some involve thousands of cysts [4]. These infection patterns are influenced by a complex interplay of parasite, host, and environmental factors, which result in the overdispersion of the larval population within the host population [9]. Both egg and intermediate host populations exhibit considerable heterogeneity. Eggs vary significantly in quantity and infectivity, while intermediate hosts display a wide range of resistance to infection [5]. A key factor driving resistance to infection is acquired immunity [6–8], yet, our understanding of this phenomenon is still incomplete.

Prior studies have suggested that animals exposed to two doses of *T. solium* eggs may not have more cysts at necropsy than those exposed to a single dose [9–11]. However, low sample sizes and heterogeneity in animal, egg viability and numbers used in experimental studies have made it hard to confirm and quantify the impact of immunity. In the most recent of those studies [9], pigs were first infected with 100,000 eggs and later reinfected with the same dose from a different tapeworm, with reinfected pigs harboring no more cysts than those infected once. However, interpretation of the results was complicated by the fact that pigs infected only with the second tapeworm developed very few cysts, making it unclear whether the reinfected pigs were truly resistant or if the second batch of eggs had lower infectivity. Additionally, the study only examined 2 kg of muscle per pig, potentially misrepresenting the actual cyst burden [9]. There is therefore a need to build on and improve on this early literature with a study design that will address most or all of the limitations of prior studies.

Greater certainty regarding immunity mechanisms may further feed into mathematical models of *T. solium*, which are often employed to simulate disease transmission. In these models, the variability in parasite burdens is often simulated through scenarios where pigs are infected at low and high intensities, sometimes with infection burdens represented by a categorization of pigs between “lightly” and “heavily” infected pigs [12–15]. Those models generally do not integrate any immunity mechanism, and simulated heterogeneities in pig infection loads may be driven by geographical dispersion of eggs, a phenomenon that remains insufficiently understood, despite recent advances [16]. However, accurate simulation of how pigs with different profiles (age, past exposure) get infected with different parasite loads – and particularly the impact of immunity in disparities in cyst burdens – is critical to developing better informed models and more accurately simulating the impact of possible disease control mechanisms.

Previous studies by our team have looked into geographical dispersion mechanisms for eggs [16] and the effect of host age as a determinant of variation in infection burden [17]. In the present study, we focused on assessing the role of acquired immunity by infecting and reinfecting pigs in highly controlled conditions and evaluating the total cyst burden across the entire carcass. Additionally, we integrated these results with our earlier findings on innate immunity to develop a model that predicts cyst numbers as a result of infection dose and age at infection. This study aimed to provide a comprehensive and quantified description of the mechanisms by which the host might regulate parasite populations.

## Methods

This study investigates: (1) the effect of reinfection on the development of cysticercosis following an initial infection with varying doses of *T. solium* eggs, and (2) the influence of a primary infection with different egg doses on the number of cysts that develop in infected pigs. These analyses are part of a broader investigation, which also examines the effect of pig age on cyst development (reported in a separate manuscript [17]). Since some pigs contributed to multiple analyses, we begin by detailing the overall experimental design before describing the statistical methods applied to address the two objectives outlined above.

### Animals

**Figure 1:**
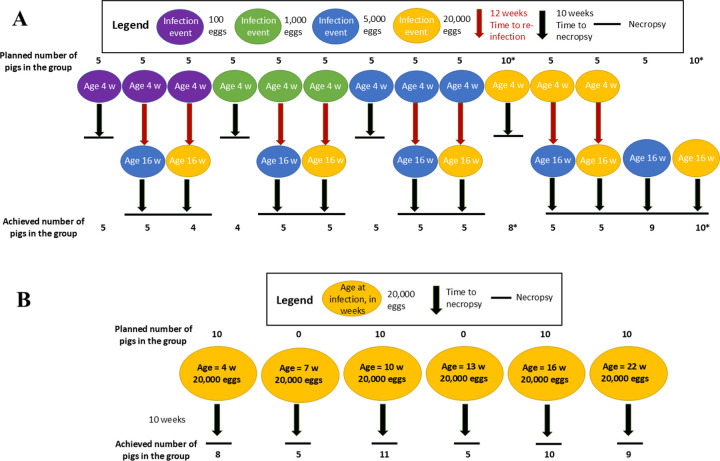
Experimental design of the study, indicating the dose, age at infection/reinfection, number of animals per group planned and achieved, and necropsy. The groups are subdivided as follows: A. Infection and reinfection design: This includes 14 groups of pigs infected with four different doses (corresponding to group “clusters”): 100, 1,000, 5,000, and 20,000 eggs given at four weeks old. Each of the doses is represented in a different color. Each cluster was divided into three groups: one necropsied after 10 weeks and the other two reinfected with 5,000 or 20,000 eggs at 16 weeks old. Two additional groups were infected once at 16 weeks old with each of the reinfection doses, representing reinfection controls. Groups marked with * were also used in the age experiment described in panel B. B. Age experiment: Pigs were infected once with 20,000 eggs at six different ages: four, seven, 10-, 13-, 16-, and 22-weeks old A complete description is available in [17].

A total of 116 pigs were purchased from 13 commercial farms in northern Peru. Pigs raised in such commercial farms are expected to be unexposed to *T. solium*. Farms were selected based on the availability of pigs within the required age range and the capacity of our corrals to accommodate them. Six pigs were excluded from the study: one had an aberrant serologic response at baseline while five died prior to the scheduled necropsy, leaving a final cohort of 110 pigs. These pigs were divided into 18 groups to address two primary objectives: (1) to evaluate the effects of acquired immunity by subjecting different groups to varying infection and reinfection doses, along with their respective infection controls (14 groups described in Fig. 1A, this study); and (2) to assess the effects of innate immunity by infecting pigs of different ages with a fixed dose (six groups described in Fig. 1B, also reported in a previous study [17]). Two groups were common for both experiments.

In the present study, we first describe and analyze the effect of reinfection. This is followed by a complementary analysis aimed at predicting the number of viable cysts based on dose, as well as other relevant factors such as sex, age, effect of reinfection, and viability of the egg pool administered.

The animals were housed in individual pens at the specific pathogen-free facilities of the Center for Global Health at Cayetano Heredia University (UPCH) in Tumbes. They were fed exclusively with properly packaged commercial feed, stored at our facilities to prevent contamination, while water was provided *ad libitum*.

Serological testing In line with the method described in [17], all pigs underwent serologic screening with antigen ELISA (TsW8/TsW5 mAb set) [18] and lentil lectin-bound glycoprotein enzyme-linked immunoelectrotransfer blot assay (LLGP-ETIB) [19] to rule out *T. solium* exposure prior to purchase. To be included in the study, pigs had to be negative on both tests (optical density ratio < 1 on Ag-ELISA and absence of any reactive band on LLGP-EITB). The mothers of all animals (except three due to owner refusal) were screened to check that they were also negative. We took weekly blood samples throughout the experiment to monitor the evolution of the serological response using both tests.

### Preparation of Egg Pools

In line with the method described in [17], tapeworms for this experiment were obtained from human stool collected in community settings as part of control interventions. Stool was collected during bowel preparation prior to antiparasitic treatment. It was screened for *T. solium* taeniasis using a combination of microscopy, coproantigen ELISA, and molecular confirmation of species using PCR [20]. Gravid proglottids were collected and stored in a saline solution at 4°C for up to two weeks. This design ensured that the eggs used in this experiment had not been exposed to any antiparasitic drug or preservative. Our study consisted of three waves of infection and reinfection, each comprising two rounds: one for the initial infection and another for reinfection, totaling six rounds. For each round, we created a pool of *T. solium* eggs from at least three different tapeworms (each coming from a different donor from the North-West of Peru – Piura and Tumbes regions) to minimize variability between infections due to differences in egg viability. The exact number of tapeworms used to create the pool utilized in each round (10, 3, 7, 8, 6, 10, and 3 respectively) varied depending on tapeworm availability and the number of viable eggs available for each tapeworm.

Egg viability was assessed for each proglottid and for the entire pool using Evans blue stain before mixing the eggs harvested from the gravid proglottids obtained from the tapeworms used in each pool. We also assessed the percentage of activated oncospheres in each proglottid and pool using the enzyme method and movement of the hexacanth embryo [21]. The percentage of activated oncospheres was 82%, on average, across all pools (range 63–97%).

### Infection procedure and Necropsy

Infections and reinfections were administered using an esophageal catheter, following a method previously described in [22] with slight modifications described in [17]. The assigned dose of *T. solium* eggs, suspended in olive oil and encapsulated in gelatin capsules, was delivered to each pig through an esophageal catheter. The capsules were flushed into the pigs’ stomach using 20–100 mL of mineral water. An intramuscular combination of ketamine (20 mg/kg) and xylazine (2 mg/kg) was used to anesthetize the pigs. After the procedure, a veterinary team monitored the pigs for vital signs and for any signs of emesis or capsule regurgitation. The reinfection procedure was performed 12 weeks after the initial infection.

We kept all pigs in individual pens for 10 weeks following the last infection, until necropsy. For necropsy, pigs were anesthetized as per the procedure described above then euthanized via intravenous sodium pentobarbital (100 mg/kg). Trained personnel then meticulously examined all muscles, the brain and other organs using 3–5 mm slicing to obtain accurate cysts counts [23]. The team identified and counted cysts and recorded the number, type (viable or degenerated) and location of cysts individually for each pig.

### Experimental designs

#### Infection-reinfection experiment

A total of 65 piglets (four weeks old) and 19 pigs (16 weeks old) were used in the infection-reinfection experiment, corresponding to the groups described in Fig. 1A. Initially, the four-week-old piglets were divided into four clusters of 15 each and infected with 100, 1,000, 5,000, or 20,000 *T. solium* eggs, respectively. After 10 weeks (the time necessary to get fully developed cysts [24]), each cluster was subdivided into three groups of five pigs: One group was necropsied to determine the cyst burden resulting from a single infection and was considered as a positive control of single infection at four weeks old. The remaining two groups were reinfected after two weeks, at 12 weeks of age, with either 5,000 or 20,000 eggs, respectively, to evaluate the additional cyst burden following reinfection with each of these reinfection doses. The remaining five piglets were also infected with 20,000 eggs and correspond to the group included in the age-related infection experiment, in which a total of 10 pigs were used.

In addition to the piglets, 16-week-old pigs were included as secondary positive controls for single infection at that age and to verify the effectiveness of the egg pool used during reinfection. These control pigs were infected once with either 5,000 or 20,000 eggs at the same time as the reinfected subgroups received their second infection. All animals infected at 16 weeks of age, whether initially infected or reinfected, were necropsied 10 weeks later, at 26 weeks of age. Additional details of each experimental group are provided in Table 1.

Due to logistical and operational constraints, the experiments were carried out in three waves of infection, each spaced approximately six months apart. Each wave included two rounds: an initial infection round, where all pigs in that wave were exposed to *T. solium* eggs, followed by a reinfection round, where a subset of the previously infected pigs was exposed again. A new pool of *T. solium* eggs was used for each infection round to ensure consistency across exposures (see Preparation of Egg Pools for details). This design allowed for a controlled evaluation of infection and reinfection dynamics.

#### Quantitative Dose-Response

We conducted multiple analyses of the number of cysts developed by pigs as a function of the infective dose. First, we considered only pigs infected once at 4 weeks with varying doses. In a second stage, we incorporated pigs infected twice (at 4 and 16 weeks) and used the results of the analysis of the effects of prior infection on pigs’ response to infection to combine those pigs with those that were infected only once. Finally, we included all pigs, regardless of their age at first infection, using evidence on the effect of age, as described in a previous paper [17], to build a comprehensive model.

#### Data analysis

For descriptive statistics, the median, minimum, and maximum values were used to summarize data. To analyze differences between infection and reinfection, a negative binomial regression model was developed, with the number of live cysts as the response variable and the infection doses at 4 weeks old and 16 weeks old as predictors, respectively. Other covariables, such as sex, the pigs’ farm of origin, the infection round (to consider possible variations between infection periods) and pool viability, were included to identify potential confounding effects.

A negative binomial regression model was also developed to create a quantitative dose-response model using data on the number of live cysts that developed following a single infection at different doses. This model was then expanded, incorporating additional animals with multiple infections and infected at different ages, by including the effect of age as described in a prior manuscript.

Model fit was evaluated using criteria such as Akaike Information Criterion (AIC) and Bayesian Information Criterion (BIC), and the best-fitting model was selected based on these indices and residual diagnostics. All the analyses were performed using R [25] and the packages MASS [26], ggplot2 [27] and plotly [28].

## Results

### Infection-Reinfection Experiment

**Figure 2:**
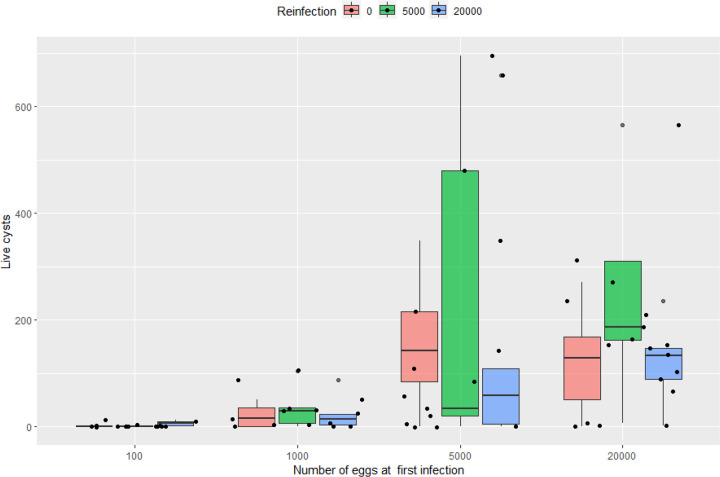
Number of live cysts (y-axis) found in pigs infected at 4 weeks old with four different doses (x-axis: 100, 1000, 5000, and 20000 eggs) [RED BAR], compared with pigs infected with the same initial dose and reinfected with 5000 eggs [GREEN] or 20000 eggs [BLUE], after 12 weeks.

The median, minimum, and maximum numbers of viable, degenerated, and total cysts observed in each of the 14 groups— either infected once or reinfected—are summarized in Table 1, along with the number of pigs per group. We found that reinfected pigs consistently exhibited similar cyst counts compared to pigs infected only once at different doses (Fig. 2). To compare cyst counts between the major clusters (pigs infected once versus pigs reinfected with 5,000 or 20,000 eggs) across the four different initial doses (logarithmic transformed), a negative binomial regression model with a two-way factorial 4 × 3 design (four different doses at four weeks old: 100, 1000, 5000, 20000 vs three clusters: infected once, reinfected with 5000 and reinfected with 20000) was applied using data from 12 of the groups (Table 2). Covariables such as sex, pool viability and/or dose adjusted for pool viability were tested but did not significantly improve the model. The analysis showed no statistically significant differences in cyst counts between pigs infected once at 4 weeks old and those reinfected with either 5,000 or 20,000 eggs, regardless of the initial infection dose, including cases where pigs initially infected with as few as 100 eggs were later reinfected with 20,000 eggs.

Two groups of pigs, infected once at 16 weeks of age with either 5,000 or 20,000 eggs, served as controls for the reinfection process. Both groups developed a substantial number of cysts (median: 43 live cysts, 90 total for 5,000 eggs and 118.5 live cysts and 286 in total for 20,000 eggs). Those cyst counts were similar to those of pigs infected at 4 weeks old with the same doses. This suggests that the eggs used in this second infection had infective potential (at least when used to infect naïve pigs of the same age) and that, should there be no immunity or partial immunity after a first infection, the statistical analysis would have been able to capture the difference in cyst numbers between pigs infected once at four weeks of age and pigs infected twice (see Table 1 for the full summary statistics of the controls cluster).

While degenerated cysts are of lesser importance (given that they do not contribute to transmission), we also sought to evaluate whether the number of degenerated cysts was higher in reinfected pigs than in pigs infected once. We therefore conducted a Kruskal-Wallis to compare both the number and the proportion of degenerated cysts. Neither comparison showed statistical significance (Kruskal-Wallis H-test: H = 2.14, *df* = 2, P = 0.34 for the number of degenerated cysts; H = 1.04, *df *= 2, P = 0.59 for the proportion of degenerated cysts).

### Quantitative Dose-Response

In the previous analysis, the dose given to pigs at first infection had a high significant association with the number of viable cysts observed after infection (Table 2), contrary to reinfection status or dose. To describe the relationship between dose and the number of viable cysts, a quantitative dose-response model was developed using negative binomial regression. A first model (referred to as the “Single Model”) includes only pigs infected once at four weeks of age and analyzes the relationship between the number of viable cysts and the infection dose. Its results are provided in the first lines of Table 3 below and depicted in Fig. S1 in supplements, where it is represented in pink.

Since no significant differences were observed between infection and reinfection (Table 2), reinfection data were combined with data on pigs infected once into a second model (the “Reinfected Model”). This model only includes pigs initially infected at 4 weeks of age. This model preserved the trend of the initial single-infection model while reducing the width of the confidence intervals, enhancing the precision of predictions.

Finally, based on findings from a previous manuscript that described the effect of age and included both an inverse age effect and a term proportional to age [17] a comprehensive model, referred to as the “exposure response model” was developed. In the “age” model, pigs infected at younger ages are less susceptible than those at the natural weaning age (approximately nine weeks old), with susceptibility then progressively decreasing as age increases. The “exposure response model” incorporated data from all pigs infected during the experiment, using both dose and age at infection as predictors. The coefficients of this model are presented in Table 3 (bottom section). The coefficient for the logarithm of the first infective dose is similar to what it was in each of the prior models and the confidence interval is similar to that of the “Reinfected” model. The blue curve in Fig. S1 represents the predicted values from this model for pigs infected at four weeks of age.

### Serological results

Results were similar to those already described for pigs involved in the “age” experiment in [17]. Briefly, all pigs used in this experiment initially tested negative on the LLGP-EITB and had antigen optical density ratios below 1. After infection, all pigs seroconverted on the LLGP-EITB between three- and nine-weeks post-infection (median: five weeks). Pigs also seroconverted on the antigen test (this consistently happened before seroconversion on the LLGP-EITB) between one- and nine-weeks post-infection (median: three weeks).

### Cysts in the brain

Out of the 110 pigs necropsied during the experiment, 16 had cysts in the brain. One of those harbored six degenerated cysts and 34 live cysts; the remaining animals presented only live cysts. Among the animals with only live brain cysts, six had only one cyst, another six had two cysts, two animals had three cysts, and one animal had nine cysts. The pig that developed 34 live cysts was infected with 5,000 eggs and reinfected with 20,000 eggs.

## Discussion

In this controlled experimental infection, we aimed to determine the impact of prior exposure to *T. solium* eggs and to assess how the number of viable cysts in pigs infected twice compares with the number of such cysts developed by pigs infected only once. This study addresses several limitations of previous studies on experimental infections with taeniids by implementing measures to reduce variability. Specifically, we aimed to ensure greater uniformity and control over host and parasite populations, as well as egg doses, to improve reproducibility and comparability of results. The present study overcame those challenges by using tapeworm pools with controlled infectivity, larger numbers of pigs, and whole-carcass cyst counts. We did not seek to control for pig genetic background as having only pure-bred animals in our experiment would have increased homogeneity but decreased the generalizability of our results, given that the general practice in commercial farming worldwide is to use mixed-breed pigs like the ones in our experiment. The study, despite its tight control of most sources of variability, still found substantial differences in infection outcomes within groups (which was to a certain extent expected [5]) but was nevertheless able to demonstrate the existence of a strong acquired immunity that effectively protects pigs from reinfection. While the exact timing of immunity development remains unclear, our results indicate that the period during which pigs remain susceptible is relatively short in relation to their overall lifespan (less than 12 weeks – as the pigs in our experiment were reinfected after 12 weeks).

The effect of acquired immunity had been hypothesized previously for taeniids [6] and several reviews had discussed how primary infections can block subsequent exposures, with multiple authors examining this phenomenon [5, 7, 8]. Although some experimental studies had reported contradictory results, such as calves being reinfected with *T. saginata* 10 weeks after the first infection, most experiments had produced opposite outcomes [29]. The main conclusion from these investigations had been that a form of acquired immunity likely develops following initial exposure [5–8, 10, 30]. These findings ultimately led to the development of the first successful vaccine against a parasitic infection: *T. ovis*, in sheep, the intermediate host [31]. There are now also commercially available vaccines against *Echinococcus granulosus* and *T. solium* [31, 32].

Our study contributed to this body of knowledge by demonstrating that immunity acquired during the initial *T. solium* infection is highly protective against infection with subsequent exposure, at least in the timeframe we evaluated. Unlike vaccines, which prime the immune system by exposing it to the TSOL18 oncosphere protein before an infection occurs [31], immunity in our study was triggered by actual exposure to eggs.

As a consequence of the required timeframe to verify reinfection effects at necropsy—given that cyst development takes approximately 10 weeks [24]—the interval between infection and reinfection in this study was necessarily long. Therefore, we could not determine the minimum time required for acquired immunity to become fully active and block reinfection. Studies on other tapeworm species suggest that this occurs progressively. For instance, experiments with *T. hydatigena* in sheep have shown that partial resistance can develop as early as seven days post-infection, with complete immunity established by 14 days [7, 33].

Another important consideration is the minimum infective dose required to trigger an effective immune response. While our results demonstrate that as few as 100 eggs can block a reinfection with 20,000 eggs, the precise threshold remains undetermined. Studies on *T. hydatigena* and *T. ovis* suggest that an effective acquired immunity can be achieved with as few as 10 eggs [34], indicating that even minimal exposure may be sufficient to induce protection. Further experiments would be needed to confirm whether this is also true with *T. solium*.

This study also showed how cyst burden relates to the egg dose at first infection. As expected, the size of the initial egg dose was found to be a significant positive predictor of the number of viable cysts, enabling the development of a model to predict live cyst numbers. Similar correlation with dose has been reported with other tapeworms and a rate of 1% of egg success was proposed for *T. solium* [7, 10]. However, our results indicate that the relationship between infection dose and the number of viable cysts is best described by a power-law pattern, rather than a strictly linear one. Specifically, the expected number of cysts increases with the dose raised to the power of 0.88, suggesting a sublinear relationship. While this estimate implies that the proportion of eggs developing into cysts may decrease at higher doses—consistent with the idea of reduced infection efficiency—we note that the 95% confidence interval includes 1, meaning this trend is biologically plausible but not statistically conclusive. This interpretation aligns with prior observations that extremely high doses (e.g., 100,000 eggs) do not produce proportionally more cysts [22]. Such sublinear dynamics complicate the explanation of superinfected pigs found in natural settings, where individuals harbor more than 10,000 cysts [35], raising the possibility that additional biological or ecological mechanisms may contribute to extreme infection outcome.

We sought to validate our exposure-response model against data from a previous study that compiled results from multiple experimental infections conducted by different research groups worldwide [35]. The model’s predictions aligned well with these independent datasets (considering expected variability in individual pig burden), further supporting its ability to describe *T. solium* cyst development across various experimental conditions. This analysis is presented in supplementary material (Fig. S2). In particular, the prior study suggested that pigs infected with 100 eggs had a [62–100%] probability of developing cysts, a range consistent with our experimental findings, where 11 out of 14 pigs (79%) receiving a first or single infective dose of 100 eggs developed cysts [35].

The results described in this paper, combined with those described in our earlier paper on the effects of age at infection [17], allowed us to leverage this unique experimental dataset to develop an overall exposure-response statistical model predicting the number of live cysts in pigs depending on both the dose of the primary infection and the age at infection. The model performed well in our dataset, capturing the observed effects of both dose and age on cyst burden.

Significant variability in cyst counts is a common observance in experimental infections with *T. solium* and other tapeworm species. We implemented several measures in an attempt to limit sources of variability in our study, including serological monitoring before, during, and after infection; housing pigs in individual pens throughout the experiment; infecting them with pools of several tapeworms using oroesophageal catheters; maintaining a controlled environment to minimize exposure to insects and other pests; and exclusively using properly stored commercial feed. Despite these precautions, we observed significant variability among infected animals, even within the same group. This outcome is not novel and is a characteristic commonly observed in experimental infections with this and other tapeworm species [5, 7]. In our study, the negative binomial dispersion parameter (θ) was estimated at 0.43 (Table 3), indicating a high level of overdispersion in cyst counts. This suggests that infection burdens vary substantially among pigs, with a greater variance than expected under a Poisson distribution. The substantial variability in the infection patterns of pigs in endemic areas could be explained in part by this infection pattern. It is also likely that pigs encounter high parasite loads when ingesting proglottids and lower loads when ingesting eggs. Finally, mechanical vectors may play a role in egg dispersion, however, studies in endemic areas have not confirmed a significant impact from this mode of transmission [16].

## Conclusions

Initial exposure of pigs to *T. solium* eggs provides strong acquired immunity against infection upon subsequent re-exposure, with protection evident even when the initial dose is as low as 100 eggs and the reinfection dose as high as 20,000 eggs. Despite minimizing variability during infection—using eggs with high in vitro viability, pooling eggs instead of entire proglottids, accurately counting eggs in infective doses, and administering infections intra-esophageally—significant variability in cyst counts was still observed within groups, suggesting that other factors may influence individual responses. Using data from these experiments, we developed a quantitative dose-response model to predict the number of live cysts in infected pigs based on egg exposure dose and age at infection. This model provides essential insights for incorporating pig immunity processes into CystiAgent [36–39], our agent-based model that simulates *T. solium* transmission and associated human neurocysticercosis model, CystiHuman [40], and evaluates the impact of control interventions in rural northern Peru.

## Figures and Tables

**Table 1:** Median, minimum, and maximum counts of live, degenerated, and total cysts across the different groups involved in the infections at 4 weeks of age, reinfections at 16 weeks of age, and controls (infected only once at 16 weeks of age), along with the corresponding number of pigs per group.

Clusters	Infection at 4 weeks	Infection at 16 weeks	Number of pigs	Live cysts			Degenerated cysts			Total Cysts		
				Median	Min	Max	Median	Min Max		Median	Min	Max
Pigs infected once	100	0	5	0	0	1	0	0	7	1	0	8
	1,000	0	4	15.5	1	51	6	3	31	33	4	59
	5,000	0	5	143	0	348	7	3	158	163	88	355
	20,000	0	7	103	0	270	63	5	490	192	71	514
Pigs reinfected with 5000 eggs	100	5,000	5	0	0	2	22	0	197	22	0	198
	1,000	5,000	5	29	1	105	4	0	15	29	8	110
	5,000	5,000	5	34	0	695	1	0	15	35	0	710
	20,000	5,000	5	187	7	565	55	1	490	199	132	731
Pigs reinfected with 20000 eggs	100	20,000	4	6	0	12	15	0	33	25.5	1	36
	1,000	20,000	5	14	4	88	15	2	34	36	16	103
	5,000	20,000	5	58	0	658	22	2	244	111	22	699
	20,000	20,000	5	134	2	236	30	0	187	189	119	238
Controls	0	5,000	9	43	0	205	28	4	555	90	14	556
	0	20,000	10	118.5	1	809	58.5	1	260	286	30	833

**Table 2:** Negative binomial regression model describing the cyst burden as a function of the log of the infection dose at 4 weeks of age and dummies reflecting single infection vs. reinfection at 16 weeks with either 5,000 or 20,000 eggs. The table includes parameter estimates, standard errors, z-values, p-values, and 95% confidence intervals for each variable in the model.

Variable	Estimate	Standard Error	z-value	p-value	95% Confidence Interval	
					Minimum	Maximum
(Intercept)	−3.59	0.88	−4.06	0.00005	−5.73	−1.28
Log (Infection dose at 4 weeks)	0.92	0.10	8.92	< 0.000001	0.65	1.18
Reinfected at 16 weeks with 5,000 vs infected once	0.57	0.49	1.18	0.24	−0.38	1.53
Reinfected at 16 weeks with 20,000 vs infected once	0.59	0.49	1.21	0.23	−0.41	1.62

**Table 3:** Negative binomial regression model describing the cyst burden as a function of the log of the infection dose. The “Single model” only includes data from pigs infected once at 4 weeks of age with different doses, the “Reinfected model” also includes data from pigs reinfected once after initial infection at 4 weeks old. Finally, the full combined “Exposure response model” also includes information from pigs infected at different ages. The table includes parameter estimates, standard errors, z-values, p-values, and 95% confidence intervals for each variable in the model.

Variable	Estimate	Standard Error	z-value	p-value	95% Confidence Interval	
					Minimum	Maximum
**Single model (only includes pigs infected once at 4 weeks of age) -** Theta (θ) value: 0.42; standard error: 0.13						
(Intercept)	−4.58	1.45	−3.17	0.0015	−8.23	0.44
Log of Infection dose	1.04	0.17	5.97	< 0.000001	0.55	1.51
**Reinfected model (includes all pigs infected at 4 weeks of age) -** Theta (θ) value: 0.43; standard error: 0.08						
(Intercept)	−2.97	0.82	−3.60	0.0003	−4.83	−0.87
Log of Infection dose	0.89	0.10	8.63	< 0.000001	0.64	1.14
**Exposure response model (includes all pigs) -** Theta (θ) value: 0.43; standard error: 0.05						
(Intercept)	0.56	1.91	0.29	0.77	−3.95	4.97
Age	−0.21	0.07	−2.87	0.004	−0.37	−0.04
Inverse of age	−10.21	5.26	−1.94	0.05	−22.10	1.72
Log of Infection dose	0.88	0.10	8.90	< 0.000001	0.63	1.10

Covariates farm of origin, the infection round, sex and pool viability were tested but were not significant.

## Data Availability

The data collected for this study are available from the corresponding author upon request.
